# Exploring the Parasite Biodiversity in Climbing Perch (*Anabas testudineus*) in Buriram, Thailand: Morphological-Based Characterization

**DOI:** 10.1155/tswj/6691770

**Published:** 2025-10-23

**Authors:** Supamas Sriwongpuk, Surachai Techaoei

**Affiliations:** ^1^Faculty of Agricultural Technology, Rajamangala University of Technology Thanyaburi, Pathum Thani, Thailand; ^2^Research Unit for Technology and Innovation for High-Value Bioproduct Production, Rajamangala University of Technology Thanyaburi, Pathum Thani, Thailand; ^3^Faculty of Integrative Medicine, Rajamangala University of Technology Thanyaburi, Pathum Thani, Thailand

**Keywords:** *Anabas testudineus*, climbing perch, ectoparasites, endoparasites, parasites

## Abstract

*Anabas testudineus* is an amphibious freshwater fish species native to Thailand, which is afflicted by protozoan and metazoan infections. This study was aimed at identifying the presence of parasites in *A. testudineus* from Thung Lam Natural Reservoir in Buriram Province, Thailand, between November 2022 and January 2023. The analysis of 120 climbing perches indicated a 100% prevalence of parasitic infections. Four species from three phyla and four genera were detected: *Trianchoratus aecleithrium*, a monogenean trematode found on gill filaments, and three intestinal parasites: *Pallisentis nagpurensis* (Acanthocephala), *Allocreadium* sp. (trematode), and *Camallanus anabantis* (Nematode). The respective prevalence rates were 100% for *T. aecleithrium*, 83.33% for *C. anabantis*, 63.33% for *P. nagpurensis*, and 26.67% for *Allocreadium* sp. Despite the widespread parasitism, no symptoms of illness were observed in the fish hosts. This investigation underscores the high prevalence of fish-borne helminthic infections in Buriram Province, Thailand.

## 1. Introduction

**Figure 1:**
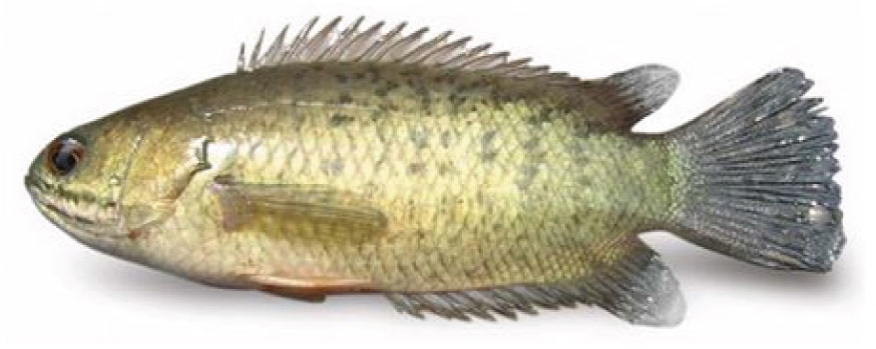
Morphology of the climbing perch (*A. testudineus*).

The climbing perch, or Pla Mor in Thai (*Anabas testudineus*), is native to Asia. The fish is also known as air-breathing labyrinth fish or walking fish because of their supplementary air-breathing organ, which allows species to survive for several days in damp conditions in addition to the water [1]. Because of their development, they have pectoral fins that allow them to maneuver themselves on the surface. The fish in question is a little freshwater species native to Asia [2], classified explicitly under the Anabantidae family and the Perciformes order. The climbing perch is anticipated to outcompete local freshwater and coastal species in population [3]. The fish has a long slender body and huge scales. The color ranges from dark to pale green to brown. The top region is dusky to olive, the underside of the head is striped, and the gill cover borders are heavily spined. The maximum size is 25 cm (Figure 1). Furthermore, when swallowed by predatory species, the fish's pointed opercular and dorsal spines lengthen [1]. According to Vidthayanon and Premcharoen [4], the fish prefers to live in canals, lakes, ponds, marshes, and estuaries. They consume algae, worms, insects, mollusks, aquatic vegetation, and small aquatic creatures; larval and juvenile fish predominantly consume plankton. They stay drowned in the moist soil throughout the dry season and hang out in pools that have jumped trees and plants [5, 6], able to keep their air-breathing organs moist and last for a few weeks or even days without water [4]. A pleasant fish food and an economically valuable food fish in Southeast Asia, climbing perch are significant fish food in that region [5].

In Buriram Province, the Thung Lam reservoir has a significant economic impact on the Nang Rong District. It functions as a natural water supply for the sake of tourism and agriculture, fishing, and irrigation, as well as providing habitat for many different species of freshwater fish [7]. Most residents catch these smaller aquatic creatures for processing into food proteins, including dried fish, fermented fish, and fish meal. However, several serious problems emerged during the culture period, primarily due to parasites, which were believed to be a significant factor in the low yield in aquaculture systems. Despite its extensive consumption in the area and importance to the community as a primary source of protein, this study was conducted to ascertain whether the parasite species infecting climbing perch (*A. testudineus*) in the Thung Lam reservoir, where it is frequently consumed by the local populace, are analogous to or divergent from those documented in other areas, especially in Buriram Province. Hence, the study was to identify and characterize the parasitic species inhabiting climbing perches, particularly those with possible adverse health effects. This work offers essential data to facilitate the surveillance and monitoring of parasite diseases in *A. testudineus*, impacting public health and aquaculture operations. The parasite records gathered in this study provide a valuable taxonomic reference for identifying and classifying parasite species associated with climbing perch and related taxa.

## 2. Methodology

### 2.1. Sampling Fish

A total of 120 climbing perch were taken from the Thung Lam reservoir between November 2022 and January 2023 for the purposes of this research. Before gathering data, fish measurements were documented, and specimens with lengths between 13 and 15 cm were chosen.

### 2.2. Collection of Parasites

Parasites were collected from fishes by dissecting and examining the skin and fin tissues using phase-contrast microscopy. Following examination using a stereomicroscope, the gills were meticulously removed and washed before being transferred to a sterile Petri plate with water to be employed in the parasite elimination treatment. Ectoparasites were analyzed by excising the gill arches from both sides of each fish and putting them in Petri dishes with 0.85% normal saline. Mucus and possible parasite substances were meticulously extracted from the gill filaments utilizing fine curved forceps. The samples were first examined using a stereomicroscope to identify ectoparasites. After parasites were found, they were separated using a micropipette, put on glass slides, and viewed under a compound microscope to determine their species and morphological characteristics. In the meantime, the object underwent a meticulous cleansing process using tap water, repeated multiple times to eliminate any extraneous substances. Initially, the mucus was taken from tiny creatures using a magnifying lens and then conducted under a microscope. In addition, following the steps described by Tonguthai et al. [8], they dealt with the parasite problem by using different strategies for each category.

### 2.3. Classification of Parasites

The descriptions of Bray [9] and Asil et al. [10] were used to identify the parasites. It was determined by distinct morphological traits unique to each taxonomic group. Identification of monogenetic trematodes focused on the morphology of the opisthaptor, encompassing the quantity and configuration of anchors, and also on the anatomy of the copulatory organ. Identification of digenean trematodes (flukes) relied on their overall morphology and the structure of the oral sucker and acetabulum, as well as the configuration and location of the testes and uterus. Classification of nematodes was based on the morphology of the buccal capsule in the anterior area. Identification of acanthocephalan was predicated on the morphology of the proboscis, encompassing the amount and arrangement of hooks, in addition to the detection and arrangement of trunk spines

### 2.4. Parasite Population Estimation

The prevalence refers to the proportion of a population that has a specific disease or condition at a particular point in time, regardless of when they first developed it, and mean intensity, in the context of parasitic infections, typically refers to the average number of parasites found within the infected individuals in a population that were assessed using the approach given by Margolis et al. [11]. Prevalence is usually expressed as a percentage. The concept or definition was the number of individuals of a host species infected with a particular parasite species/the number of hosts examined.

## 3. Results

### 3.1. The Occurrence of Parasites

Table 1 illustrates the four parasitic species, the overall parasite count, and the fish species affected in the climbing perch specimens obtained from Thung Lam Lake over the period of November 2022 to January 2023. An examination of all 120 fish samples revealed the presence of three internal parasites and one external parasite, each belonging to a different class: *Trianchoratus aecleithrium* (Monogenea), *Allocreadium* sp. (Trematoda), and *Camallanus anabantis* (Nematoda), and the species *Pallisentis nagpurensis* belongs to the phylum Acanthocephala, also commonly referred to as the spiny-headed worms.

Table 2 demonstrates the frequency and average strength of the parasites found in the climbing perch. The graph showed a 100% prevalence for *T. aecleithrium*, indicating that this pathogenic species was present in every fish sample. *Allocreadium* sp., along with *Allocreadium nagpurensis*, had prevalences of 26.67%, 83.33%, and 63.33%, respectively. The respective mean intensities were 4.17, 2.63, 2.20, and 1.79.

### 3.2. Species Classification

**Figure 2:**
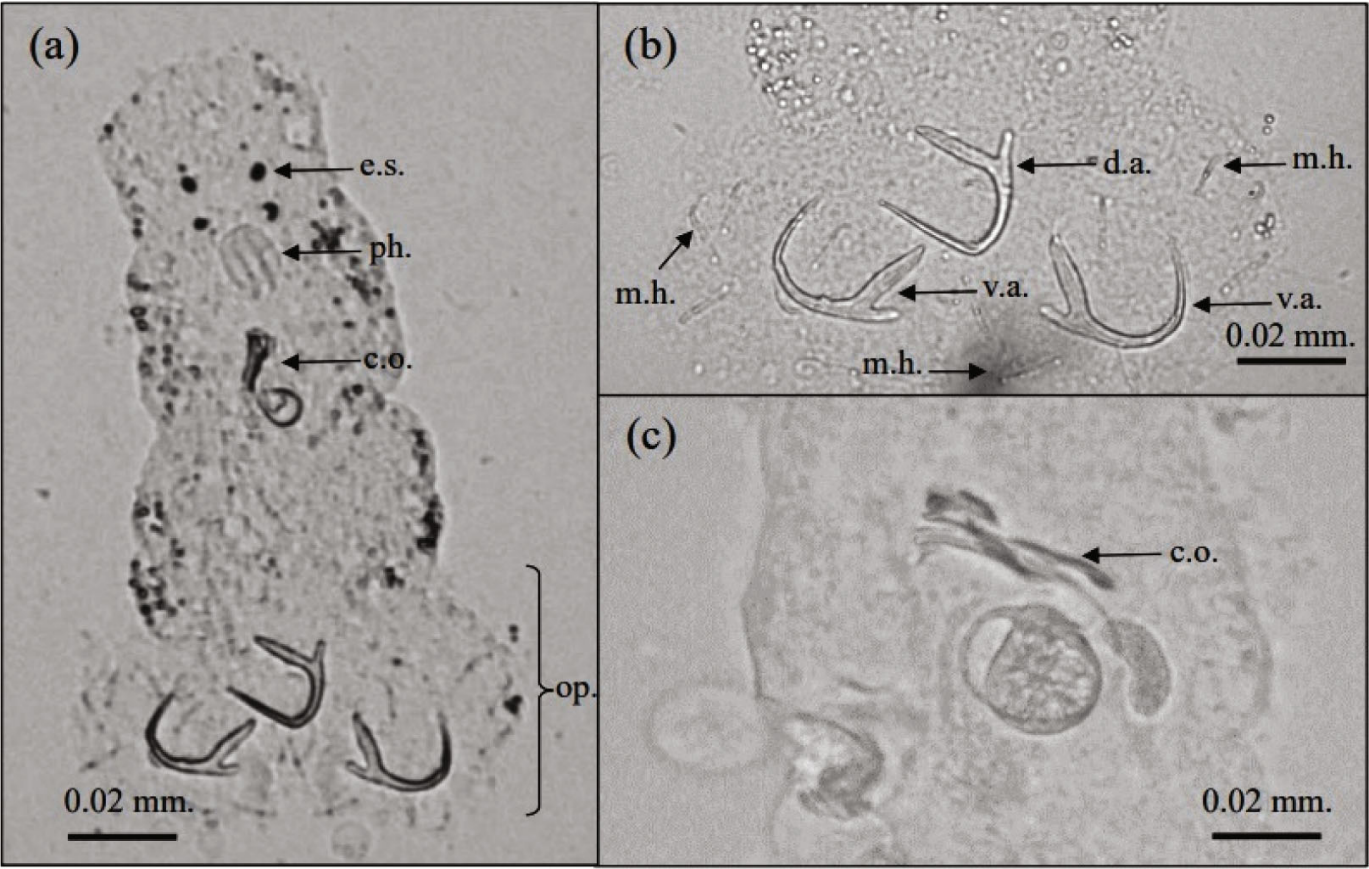
*Trianchoratus aecleithrium*. (a) The characteristic of *Trianchoratus aecleithrium*. (b) Opisthaptor characteristic of *T. aecleithrium*. (c) Male reproductive organ or copulatory organ of *T. cleithrum*. op. = opisthaptor, c.o. = copulatory organ, e.s. = eye spot, ph. = pharynx, d.a. = dorsal anchor, v.a. = vental anchor, m.h. = marginal hooks. Scale bar = 0.02 mm.

*T. aecleithrium* was a monogenean (class Monogenea, phylum Platyhelminthes), corresponding to a specific form of flatworm with a stretched and flattened shape with a top-to-bottom alignment. It was largely detected on the gill filaments of fish by researchers. An examination discovered that the actual size of the parasite varied between 0.18 and 0.29 mm in length and between 0.07 and 0.08 mm in width. The front segment of the organism had a smooth and fragile outer layer adorned with two sets of simple eyes. The pharynx, a circular formation, had a diameter ranging from 0.01 to 0.02 mm. The opisthaptor, a bilobed attachment organ, is clearly separated from the body, measuring 0.06 mm in width and 0.03–0.04 mm in length. This distinct morphology is critical for species identification. The parasite possessed three anchors: two ventral anchors with base widths measuring 0.02, 0.02, and 0.01 mm; point length of 0.01 mm; inner root length of 0.01 mm; and outer root length of 0.01 mm, and the specimen consists of a solitary dorsal anchor with three distinct base widths (0.04, 0.03, and 0.01 mm), two varying established lengths (0.01 and 0.01 mm, respectively), and neither a single inner nor outer root length. There were no support bars on the opisthaptor, though. The 14 marginal hooks were added, totaling 0.01 in length. The copulatory organ consisted of the accessory component and the nonarticulated cirrus, which had a combined length of 0.03 mm. The moderately sclerotized vaginal tube supports the lateral vaginal opening (Figure 2).

**Figure 3:**
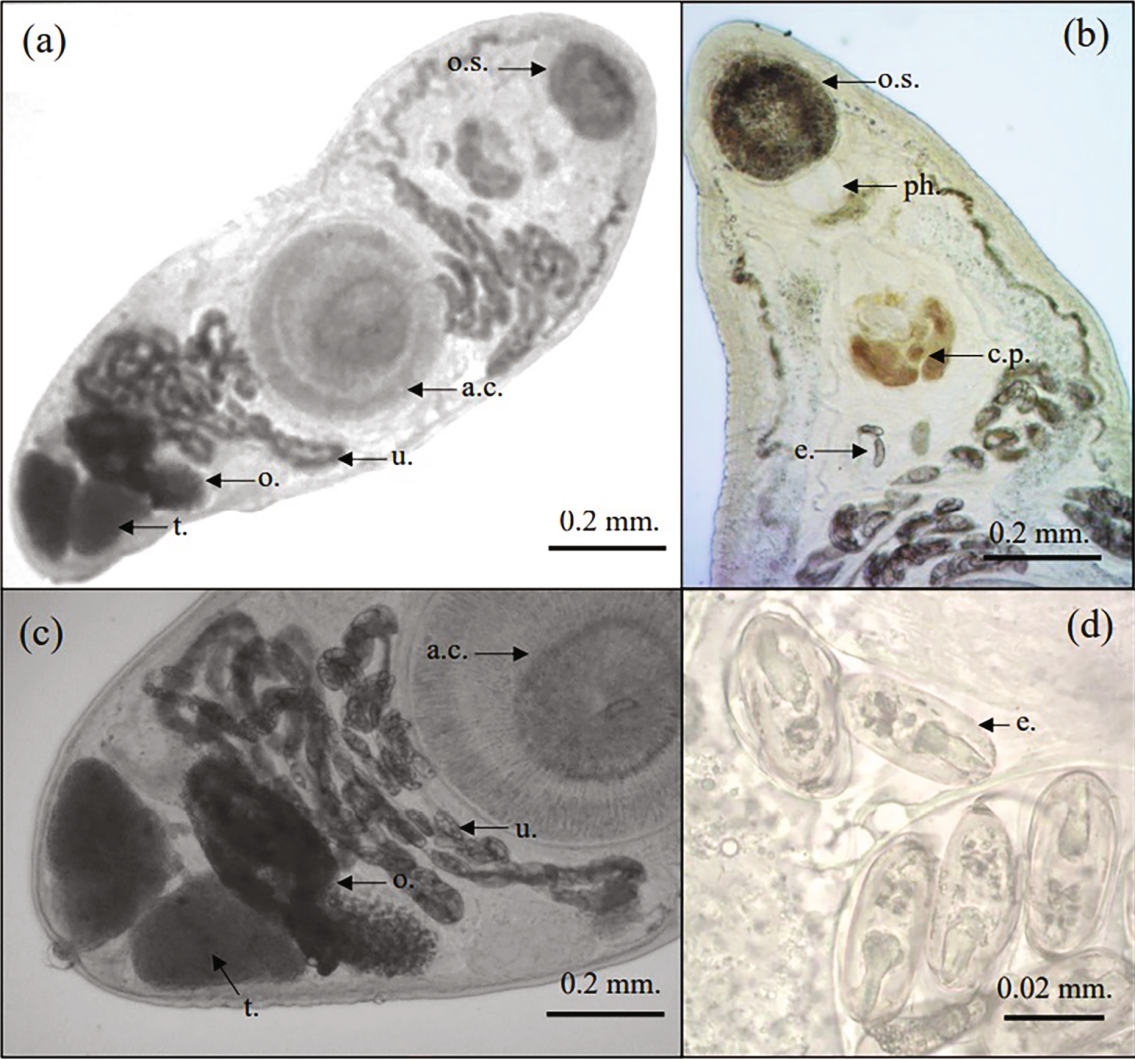
Morphology of *Allocreadium* sp. in climbing perch. (a) Adult of *Allocreadium* sp. (b) The oral sucker with a well-developed pharynx of *Allocreadium* sp. (c) Reproductive system of *Allocreadium* sp. (d) Eggs of *Allocreadium* sp. o.s. = oral sucker, a.c. = acetabulum, u = uterus, o. = ovary, t. = testes, ph. = pharynx, c.p. = cirrus pouch, e. = egg.

The intestines of fish harbor a parasitic trematode known as *Allocreadium* sp. The vertebral canal presented a stretched and compacted form, measuring 3.80 mm in length and 0.84 mm in width. The acetabulum, located anteriorly to the body, displayed the maximum width. The pharynx exhibited muscularity and had an oval-to-spherical morphology, with dimensions that measured around 0.02 mm in length and 0.01 mm in width. The anterior ceca, found on either side of the acetabulum, possessed a width of around 0.04 mm and functioned to separate the esophagus. The ceca displayed a bigger form, extensive size, and lack of visual capabilities. The posterior section was approximately 2.70-mm long and 0.04-mm thick. The acetabulum revealed an elliptical to spherical structure, exhibited dimensions of just under 5 mm in length and 0.03 mm in width, and displayed muscular grids. The oral sucker showed approximate length and diameter readings of 0.03 and 0.03 mm, respectively. The cirrus, a large spherical sac with an average length of generally 0.03 mm and a diameter of 0.02 mm, was discovered at the back part of the pharynx (Figure 3).

The testes, located in the posterior region, connected to the uterus, attached to the acetabulum, with the small, pear-shaped seminal receptacle positioned below the ovary. The ovary exhibited an elliptical shape, measuring 0.04 mm in length and 0.02 mm in width. It was smaller than the testes and protruded past the back edge of the acetabulum. The testes exhibited an extended and oval shape. The correct testis measured around 0.05 mm in length and 0.02 mm in width, while the left testis measured around 0.05 mm in length and 0.026 mm in width. The vitellaria were positioned on the ventral side of the sucker's posterior border, whereas the excretory organ's bladder exhibited a tubular morphology (Figure 3).

**Figure 4:**
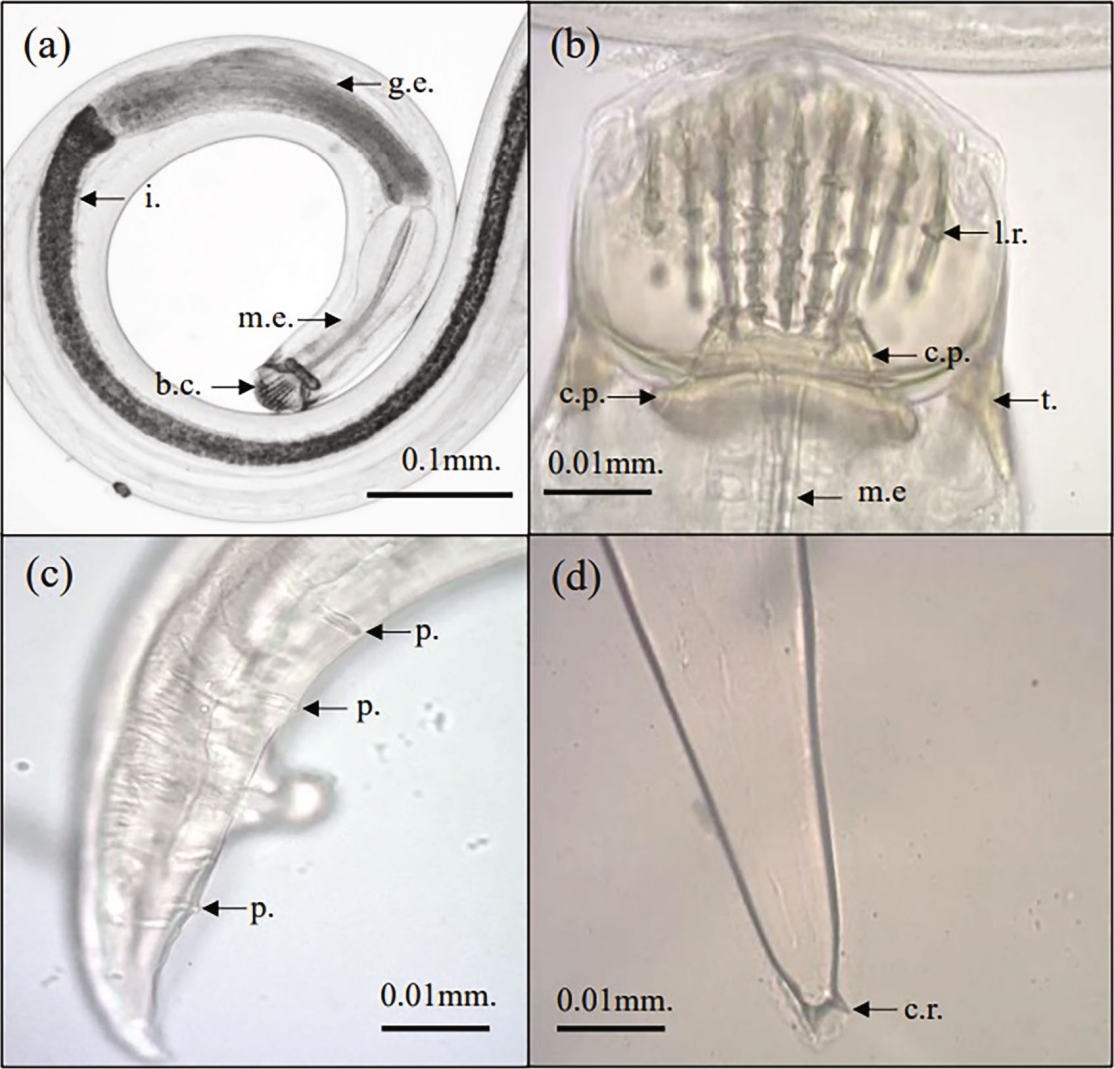
Morphology of *Camallanus anabantis*. (a) Adult of *C. anabantis*. (b) The components of the buccal capsule of *C. anabantis*. (c) The specifics of the posterior end of *C. anabantis* tail. (d) The posterior end of *C. anabantis* tail. Note: b.c. = buccal capsule, m.e. = muscular esophagus, g.e. = glandular esophagus, i = intestine, l.r. = longitudinal ridges or beaded ridge, c.p. = chitinous pharynx, t. = trident, p. = pre-anal papillae, c.r. = caudal rami.

*C. anabantis* was a small- to medium-sized roundworm or nematode (Figure 4). Essentially, the worms had a sleek outer layer covering their bodies, a mouth opening that resembled a narrow slit, and three hard, chitinous structures. Each lateral valve of the buccal capsule displayed identical characteristics, internally equipped with nine longitudinal ridges that varied in length based on the individual's gender. A robust, hardened ring known as the basal ring encircles the posterior end of the buccal cavity. Two prominent tridents, each consisting of three spikes, reinforced this ring. Anatomically, we separated the esophagus into two sections: the forward muscular and the posterior glandular. The glandular region showed a significant increase in both width and length compared to the muscular components. There is a prominent, clearly delineated abdominal region. Both males and females had an excretory opening located in the preequatorial region. The male worms possess two asymmetrical and slender spicules, characterized by a rounded anterior tip and a pointy posterior tip. Females from the postequatorial region had small lips that were slightly turned upward. We discovered ovoid-shaped eggs in the uterine cavity, positioned toward the back of the vulva. Each interval consistently revealed small bifid appendages with sharp tips.

Both sexes possessed finger-shaped mucrons at the tips of their tails. Males' lengths ranged from 3.85 to 5.83 mm, with a width of 0.10–0.18 mm. The caudal papillae consisted of two types of cloacal papillae: five sets of postanal papillae and five sets of preanal papillae. Both spicules featured similar shapes but varied significantly in size. The females had a diverse range of lengths, between 12.30 and 19.50 mm, whereas their widths varied between 0.25 and 0.50 mm.

**Figure 5:**
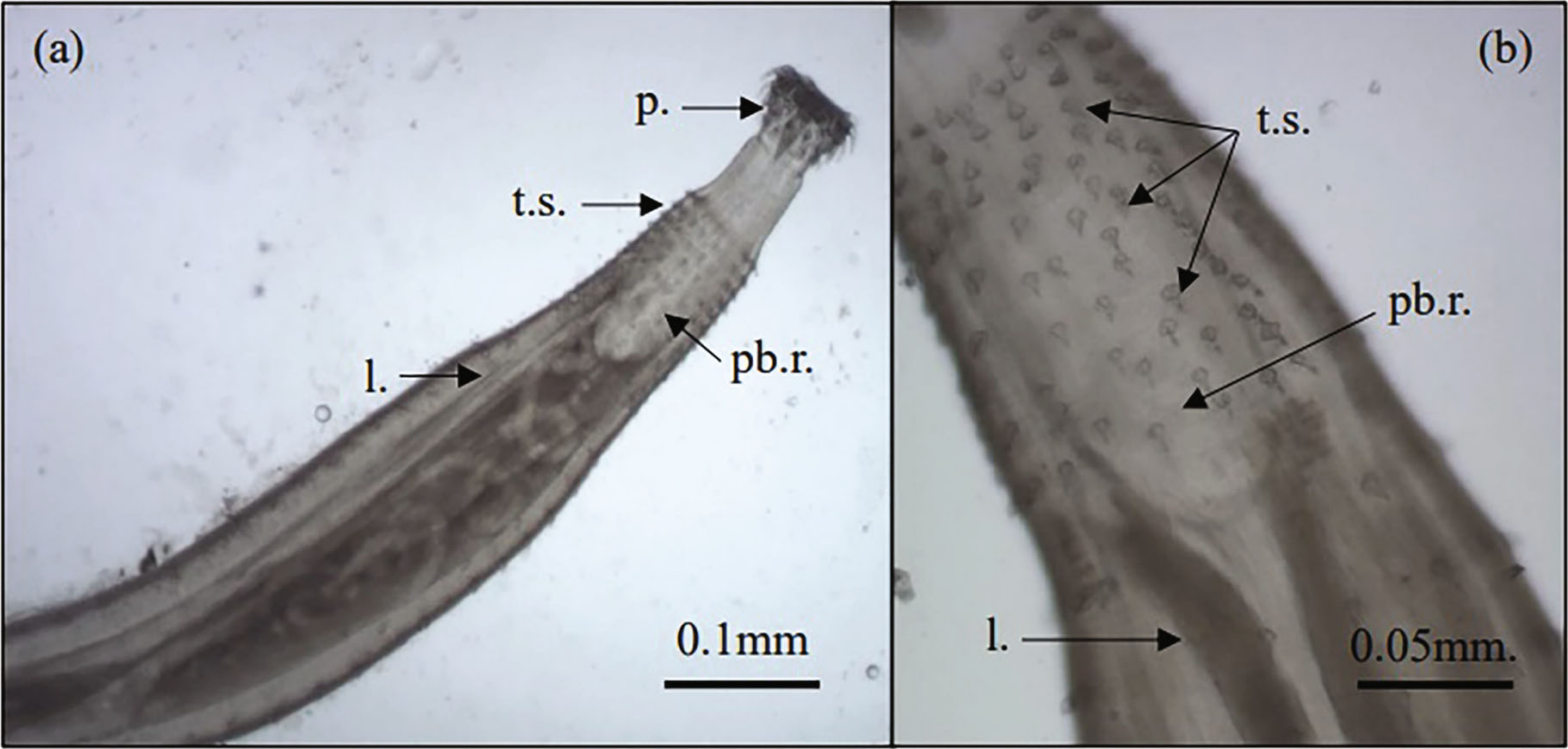
Morphology of Acanthocephala, *Pallisentis nagpurensis*. (a) Head part of *P. nagpurensis*. (b) Body skin part of *P. nagpurensis*. p = proboscis, t.s. = trunk spine, pb.r. = proboscis receptacle, l = lemnisci.

*P. nagpurensis*, the dimensions of *P. nagpurensis* were 4.1 mm in length and 0.3 mm in width, with a cylindrical structure. The parasite's proboscis, with dimensions of 0.17 mm in length and 0.2 mm in width, had a significant size and circular appearance. It was adorned with four sets of 10 slender, curved barbs of different sizes but identical in shape. The hooks in the early circle were stronger and larger than those in the basal row. As shown in Figure 5, each hook had a handle that was placed inside the proboscis wall, a horizontally oriented root, and a curved blade. The caudal papillae consisted of two pairs of cloacal papillae, five pairs of postanal papillae, and five pairs of preanal papillae. Both spicules displayed comparable shapes, although they varied in size. The females demonstrated a variety of lengths ranging from 12.30 to 19.50 mm, while their widths varied from 0.25 to 0.50 mm. Furthermore, the neck exhibited considerable elongation, while the proboscis receptacle displayed a sac-like morphology consisting of a single layer. A marginal position separates the proboscis receptacles from the lemniscal tubules. The body comprised the clavicles and spine. The organism's collar displayed 14–16 concentric patterns, with each pattern consisting of 16 spines. The trunk displayed 20 circles, each containing 14–16 spines; the anterior and posterior testes measured 3.5 mm in length and 1.5 mm in breadth, respectively. Cement gland measurements were 0.3 mm in length and 0.1 mm in diameter; seminal vesicles were 0.06 mm wide and 0.3 mm in length, and they were placed behind the testes. The reservoir, which was filled with cement, measured 0.2 mm in length and just over 1 mm in breadth. Each testis contained the articular bursa, cement gland, and cement reservoir adjacent to the vas deferens glands (Figure 5).

## 4. Discussion

Protozoan parasites were the main factors responsible for causing several infectious diseases in fish, specifically in climbing perch species. Ecto- and endoparasitic protozoa played crucial roles as highly significant organisms [12]. The distribution of protozoan reproduction varies throughout different regions of a fish body. A significant population of parasites inhabited the gills, leading to a reduction in respiratory capacity and potentially causing death [13]. This finding aligns with our previous investigation, which identified external parasites in the gill cavity of *T. aecleithrium*. In 1966, Price and Berry identified the genus *Trianchoratus* located inside the gills of the proposed gourami (*Helostoma rudolfi*), a fish species native to Southeast Asia and belonging to the Helostomatidae family. The *Trianchoratus* had three fully formed anchors. In 2017, researchers discovered *T. aecleithrium* in the gills of *Trichogaster trichopterus* [14]. The intensity and prevalence of monogeneans suggest an immunosuppressive state resulting from infestation, leading to damage of gill organs. Various histopathological alterations in the gills, including hypertrophy, telangiectasis, gill degeneration, coalescence, and hyperplasia (Grades I, II, III, and IV), were notably evident [15].

Actually, *P. nagpurensis*, an acanthocephalan, was the intestinal parasite that affected fish, amphibians, birds, reptiles, and mammals. The organism had the ability to obtain nutrients by piercing the intestinal wall of its host with its spiky proboscis. Its abundance had the ability to clog the stomach and intestinal lumens, leading to the death of the fish host [16]. According to Dekari et al. [17], parasitic diseases have the ability to change demographic traits and economic importance. Additionally, they can impact the host's body weight or ability to reproduce. However, to obtain precise and trustworthy species-level confirmation, it is advised to incorporate molecular techniques, specifically the 18S/COI sequencing, even though morphological traits are still essential for parasite identification [18].

Across different geographical regions, a diverse array of parasites and protozoa has been reported in *A. testudineus*. In India, studies have documented a high prevalence of protozoan parasites such as *Ichthyophthirius multifiliis*, a ciliate protozoan that causes white spot disease, resulting in epidermal damage and secondary bacterial infections [19]. Additionally, myxozoan parasites, commonly found on the gills and skin of *A. testudineus*, have been reported in both India and Bangladesh, especially in aquaculture systems with poor water quality [20]. These protozoans are considered opportunistic pathogens that thrive under stress conditions, leading to morbidity and mortality in affected fish populations. Metazoan parasites, particularly trematodes, also feature prominently in parasitological surveys of *A. testudineus*. In Thailand, *Centrocestus formosanus*, a heterophyid trematode, has been identified in the gills of *A. testudineus*, raising public health concerns due to its zoonotic potential. Human infections occur through the consumption of raw or inadequately cooked fish containing metacercariae. Clinical manifestations in humans range from mild gastrointestinal disturbances to more severe symptoms depending on the worm burden [21].

Similarly, in Vietnam and Laos, *Haplorchis* spp. metacercariae were recovered from *A. testudineus*, especially in regions with a high prevalence of traditional raw fish dishes. This underscores the importance of integrating fish parasitology with food safety strategies in endemic areas [22].

In Malaysia, *C. complanatum*, also known as the “yellow grub,” has been identified in the muscular tissue of *A. testudineus*. This trematode poses a hazard to humans upon consumption of raw fish, potentially resulting in laryngopharyngitis as the parasite migrates to the esophagus. Despite the rarity of human occurrences, the public health implications necessitate enhanced surveillance [23]. Additionally, finding trematode metacercariae in fish from rivers and rice fields shows that these parasites have a complicated life cycle that involves snails and birds. This ecological interaction promotes the persistence and dissemination of these parasites in freshwater ecosystems.

The global distribution of protozoan and helminth parasites in *A. testudineus* is further obstructed by the biological diversity of their habitats. Surveys in the Philippines discovered *Cryptobia* spp., flagellated protozoa present in the bloodstream, associated with anemia and systemic diseases in fish raised in intense aquaculture environments [24]. These parasites, although not zoonotic, significantly affect fish morbidity and lead to economic losses. The climbing perch (*A. testudineus*) and related anabantid species harbor a diverse assortment of endo- and ectoparasites, with composition markedly varying across geographical regions due to differences in ecological factors, host availability, and human impacts. In Southeast Asia, where their life cycles rely on appropriate intermediate hosts, such as freshwater snails, and definitive hosts, such as piscivorous birds, trematodes, such as *Allocreadium* spp., are commonly reported [25]. In contrast, African anabantid species, especially those in the genus *Ctenopoma*, often harbor endemic cestodes, monogeneans, and nematodes with limited geographical ranges, suggesting localized host–parasite coevolution [26, 31]. Environmental variables, including temperature, pH, and hydrology, such as in relation to low pH, low dissolved oxygen, and high temperature favoring parasite outbreaks, affect the development and transmission of parasite stages, whereas aquaculture techniques, including stocking density, chemotherapeutic interventions, and water source, can either hinder or facilitate the spread of infections. Moreover, genetic variability among host species and populations may confer differing degrees of resistance to infection, mediated by immunological mechanisms and molecular interactions between host and parasite [2, 27–30]. Human actions, including fish translocation and the introduction of nonnative species, have intensified the spread of parasitic taxa beyond their native ranges, often leading to the establishment of new host–parasite associations in various ecological contexts [30]. Thus, understanding the distribution of parasites in climbing perch across different biogeographical regions requires a thorough framework that combines parasitological surveys, molecular diagnostics, ecological modeling, and management strategies to mitigate risks to fish health and aquatic biodiversity.

The observations of parasite distribution and burden indicate that parasite species have significant host selectivity, especially for fish species. The current global climate crisis, especially global warming, profoundly affects water quality, resulting in alterations in pH and decreases in dissolved oxygen levels. These modifications foster conditions favorable to the proliferation and transmission of aquatic parasites, which have been demonstrated to expedite parasite life cycles, resulting in a wider and more rapid spread of parasitic illnesses; nonetheless, host–parasite specificity remains notably stable. Hence, they heighten the danger of parasitic infections and the onset of diseases in aquatic creatures [31, 32]. For instance, notwithstanding the taxonomic diversity among monogenean parasites, *T. aecleithrium* is invariably linked to *A. testudineus* (the climbing perch). Likewise, within the trematode category, despite the identification of numerous species, *C. anabantis* has mainly been documented in *A. testudineus* or other species belonging to the genus *Anabas*. These patterns highlight the robust evolutionary and ecological connections that regulate host–parasite interactions, even in the face of fluctuating environmental conditions. Parasites normally occur in both wild and farmed fish. Nevertheless, fish can be safely ingested by humans provided they are cooked adequately at appropriate temperatures to eradicate parasitic illnesses [33] or employed for medicinal significance, such as mahua oil cake extract, against external parasites, specifically *Argulus foliaceus*, in *Cyprinus carpio* (Linnaeus, 1758) [26]. This research has limitations, including the absence of morphological references for each parasite species and an insufficient amount of biomolecular data for analyzing parasite–host relationships.

In summary, *A. testudineus* serves as a reservoir for multiple protozoan and helminth parasites with both veterinary and public health implications. The following table synthesizes the findings from different countries to provide a comparative understanding of the parasitic infections reported in this species as shown in Table 3. These data clearly indicate the widespread occurrence of protozoan and helminth infections in *A. testudineus* across various regions, with certain parasites such as trematodes posing significant zoonotic threats. Integrative approaches involving fish health monitoring, ecological management, and public education on safe fish consumption are crucial for mitigating the risks associated with these parasites.

## 5. Conclusion

In conclusion, we discovered four varieties of ecto- and endoparasites in four phyla: Monogenea Trematode (*T. aecleithrium*), Trematoda (*Allocreadium* sp.), Nematoda (*C. anabantis*), and Acanthocephala (*P. nagpurensis*) in Buriram Province, Thailand. The research found that the highest levels of parasite infection were seen in the monogenetic trematode *T. aecleithrium* and the parasite *C. anabantis*. Each parasite type was closely connected to its particular fish host, showing clear host specificity. The parasite species were strongly linked to their specific fish host in these infections, demonstrating a definite host specificity. In light of the prevalence of parasitic diseases affecting fish in Buriram Province, Thailand, it is puzzling that the fish samples tested positive for four distinct parasite species without exhibiting any signs of disease. High parasite loads can indicate environmental issues such as water pollution and ecological stress, which may affect fish populations and biodiversity, highlighting the need for effective environmental management and conservation efforts.

## Figures and Tables

**Table 1:** Parasites in the climbing perch from the Thung Lam reservoir in Buriram Province, Thailand.

**Parasitic species**	**Number of fish with parasites**	**Total number of parasites**
Monogenetic trematode (*T. aecleithrium*)	120	500
Trematoda (*Allocreadium* sp.)	32	84
Nematoda (*C. anabantis*)	100	220
Acanthocephala (*P. nagpurensis*)	76	136

**Table 2:** Parasite prevalence and mean intensity of the samples from the climbing perch.

**Parasitic species**	**Prevalence (%)**	**Mean intensity**
*T. aecleithrium*	100.00	4.17
*Allocreadium* sp.	26.67	2.63
*C. anabantis*	83.33	2.2
*P. nagpurensis*	63.33	1.79

**Table 3:** The report of the prevalence of the parasite and protozoa.

**Fish**	**Species of parasites**	**Protozoa**	**Origin of fish**	**References**
*A. testudineus*	*Centrocestus formosanus*	*I. multifiliis*	Thailand	[14, 16]
*A. testudineus*	*Haplorchis* spp.	*Trichodina* spp.	Vietnam and Laos	[15, 17]
*A. testudineus*	*C. complanatum*	*—*	Malaysia	[18]
*A. testudineus*	*—*	*Cryptobia* spp.	Philippines	[19]
*A. testudineus*	*—*	*I. multifiliis* and *Trichodina* spp.	India and Bangladesh	[14, 15]
*A. testudineus*	*Acanthostomum* sp.	—	Thailand	[20]
	*Allocreadium* sp.			
	*Camallanus* sp.			
	*Centrocestus caninus*			
	*Pallisentis* sp.			
	*Stellantchasmus falcatus*			
	*Trianchoratus* sp.			
*A. testudineus*	*Haplorchis pumilio*	*—*	Vietnam	[27]
	*Centrocestus formosanus*			

## Data Availability

The data used to support the findings of this study can be obtained from the corresponding author upon reasonable request.
